# Characterization of *Anopheles stephensi* Odorant Receptor 8, an Abundant Component of the Mouthpart Chemosensory Transcriptome

**DOI:** 10.3390/insects12070593

**Published:** 2021-06-30

**Authors:** Zachary Speth, Gurlaz Kaur, Devin Mazolewski, Rayden Sisomphou, Danielle Denise C. Siao, Rana Pooraiiouby, Hans Vasquez-Gross, Juli Petereit, Monika Gulia-Nuss, Dennis Mathew, Andrew B. Nuss

**Affiliations:** 1 Cell and Molecular Biology Graduate Program, University of Nevada, Reno, NV 89557, USA; zspeth@nevada.unr.edu (Z.S.); gurlazk@nevada.unr.edu (G.K.); dmazolewski@nevada.unr.edu (D.M.); 2 Department of Agriculture, Veterinary and Rangeland Sciences, University of Nevada, Reno, NV 89557, USA; raydensisomphou1@gmail.com (R.S.); danielle.siao@gmail.com (D.D.C.S.); rpooraiiouby@med.unr.edu (R.P.); 3 Nevada Bioinformatics Center, University of Nevada, Reno, NV 89557, USA; hvasquezgross@unr.edu (H.V.-G.); jpetereit@unr.edu (J.P.); 4 Department of Biochemistry and Molecular Biology, University of Nevada, Reno, NV 89557, USA; mgulianuss@unr.edu; 5 Department of Biology, University of Nevada, Reno, NV 89557, USA; dennismathew@unr.edu

**Keywords:** odorant receptors, transcriptome, mouthparts, chemoreceptor, maxillary palpi, labellum, sulcatone

## Abstract

**Simple Summary:**

Human malaria is transmitted by mosquitoes in the genus *Anopheles*—particularly species that prefer to feed on humans. The mosquito’s sense of smell drives this preference; however, most studies have focused on species native to Sub-Saharan Africa. Malaria vectors in other parts of the world may use different odorants to choose hosts. We, therefore, focused on *Anopheles stephensi*, the south Asian malaria mosquito, in this study. Mosquitoes have different organs specialized for odor perception, such as the antennae; however, we focused on the mouthparts (primarily the maxillary palp and labella) in this study. We used the RNAseq technique to determine which odor receptors are present in the mouthparts and then focused on one of these receptors: Or8. Using a technique known as the *Drosophila* empty neuron system, we tested this receptor’s ability to detect different odorants, particularly chemicals emitted by humans. This receptor in *An. stephensi* detected similar odors to a homologous receptor in an African species, *Anopheles gambiae*, with the exception of the chemical sulcatone. This chemical is an important attractant in other disease-transmitting mosquitoes and suggests that different mosquito species may be using odors differently to find hosts.

**Abstract:**

Several mosquito species within the genus *Anopheles* are vectors for human malaria, and the spread of this disease is driven by the propensity of certain species to feed preferentially on humans. The study of olfaction in mosquitoes is important to understand dynamics of host-seeking and host-selection; however, the majority of these studies focus on *Anopheles gambiae* or *An. coluzzii*, both vectors of malaria in Sub-Saharan Africa. Other malaria vectors may recognize different chemical cues from potential hosts; therefore, in this study, we investigated *An. stephensi*, the south Asian malaria mosquito. We specifically focused on the mouthparts (primarily the maxillary palp and labella) that have been much less investigated compared to the antennae but are also important for host-seeking. To provide a broad view of chemoreceptor expression, RNAseq was used to examine the transcriptomes from the mouthparts of host-seeking females, blood-fed females, and males. Notably, AsOr8 had a high transcript abundance in all transcriptomes and was, therefore, cloned and expressed in the *Drosophila* empty neuron system. This permitted characterization with a panel of odorants that were selected, in part, for their presence in the human odor profile. The responsiveness of AsOr8 to odorants was highly similar to *An. gambiae* Or8 (AgOr8), except for sulcatone, which was detected by AsOr8 but not AgOr8. Subtle differences in the receptor sensitivity to specific odorants may provide clues to species- or strain-specific approaches to host-seeking and host selection. Further exploration of the profile of *An. stephensi* chemosensory proteins may yield a better understanding of how different malaria vectors navigate host-finding and host-choice.

## 1. Introduction

Worldwide, approximately 228 million malaria cases occurred in 2019, resulting in over 400,000 deaths [1]. In southern Asia, the predominant urban malaria vector is *Anopheles stephensi*, commonly referred to as the Asian malaria mosquito [2]. Alarmingly, *An. stephensi* was recently introduced to the Horn of Africa region, causing a spike in malaria cases and jeopardizing malaria eradication efforts there [3]. Its propensity for developing in human-made habitats, including water cisterns and wells, make it uniquely suited for an urban habitat, and the anthropophilic feeding habits of the strain *An. stephensi* facilitate a cycle of rapid and continued human–mosquito–human malaria transmission [4]. 

Despite significant progress in preventing malaria cases, particularly in the last decade, gains have plateaued since 2015, thus, suggesting limitations of the current strategies in use and that additional approaches are needed [1]. Understanding the factors that govern mosquito host-seeking and host choice is one area that may lead to strategies for preventing disease transmission.

Mosquitoes possess three main classes of chemoreceptors for detecting environmental chemicals via smell or taste: odorant receptors (Ors), ionotropic receptors (Irs), and gustatory receptors (Grs). All Ors require the pairing of an odorant receptor co-receptor (Orco) to one of multiple “tuning” Ors to function. In addition, odorant-binding proteins (OBPs) function to shuttle odorants through the sensillar lymph to the chemoreceptors in the neuronal membrane [5]. Humans emit several odorants from the breath, the skin, and the skin microbiome that are attractive to mosquitoes. These odorants include CO_2_, common to all respiring animals and attractive to host-seeking mosquitoes in general; however, specific odorants more abundant in humans are additionally attractive to human-specialist mosquitoes. For instance, specific odorant receptors are receptive to a selection of human-emitted odorants in *Anopheles gambiae* [6,7]. 

Additionally, several Ors are upregulated in the antennae of host-seeking female *An. gambiae* or *Anopheles coluzzii* in comparison to blood-fed females, males, or the cattle-preferring *Anopheles quadriannulatus* [8,9,10,11]. However, different mosquito species may be attracted to different components of the human-emitted odor spectrum. *Aedes aegypti*, for instance, is highly attracted to lactic acid, an abundant odorant emitted from human skin residues [12]. In contrast, *An. gambiae* and *An. coluzzii* are only weakly attracted to lactic acid, but instead respond more strongly to ammonia, which is abundant in human sweat [5]. Much remains to be explored regarding chemoreception and host preference in mosquito species other than model vector species, such as *An. gambiae, An. coluzzii*, and *Ae. aegypti*. The availability of additional Anopheline genomes continues to expand and facilitates the characterization of homologous odorant receptors in other species [13].

Although antennae play a strong role in mosquito chemosensation, the mouthparts (principally the maxillary palps and the labella) also have critical host-seeking roles. For instance, removal of the maxillary palps in *An. stephensi* females significantly reduced the ability of mosquitoes to find a host, similar to the reduction in the host-finding capabilities in antennae-less females [14]. We, therefore, used RNAseq to explore the chemosensory repertoire of the mouthparts of *An. stephensi* to better understand odor perception in this set of sensory structures. Among other chemoreceptors and OBPs, the odorant receptor Or8 was abundantly expressed in the mouthparts. To better understand this receptor’s role in host-seeking and selection, we used electrophysiology approaches to characterize this receptor’s response to a panel of odorants, including human volatiles. We further characterized the response of the capitate peg of *An. stephensi* maxillary palps to the odorant panel and found a similar response profile to the heterologously expressed AsOr8.

## 2. Materials and Methods

### 2.1. Mosquito Rearing

*Anopheles stephensi* colonies (STE2, MRA-128 strain, obtained from MR4, origin: Delhi, India) were maintained at 28 °C and 70% relative humidity on a 16:8 photoperiod (light:dark) in a dedicated insectary as previously described [15]. Briefly, first, the instar larvae were counted into rearing pans and fed daily on a ground fish food diet (Tetramin^®^, Melle, Germany) until pupation. The adults were provided with water and a 10% sucrose solution ab libitum for regular colony maintenance. For egg production, adult females were maintained with an artificial membrane feeder on bovine blood [16].

### 2.2. An. stephensi Mouthpart Transcriptome

#### 2.2.1. RNA Sample Collection and Sequencing

The entire complement of mouthparts, including the maxillary palpi and proboscis, were dissected from cold-anesthetized adult mosquitoes and placed in TRIzol reagent (Invitrogen, Carlsbad, CA, USA) on ice. We collected 500 mouthparts per sample. For females, one group was blood fed 12 h prior to mouthpart collection, while the other female group and males were maintained on 10% sucrose only, as per normal colony conditions. Due to limited resources, only single replicate collections were made for each condition. All mosquitoes were 5-days post-emergence, and all collections were performed at dusk, approximately 1–2 h before scotophase. Over 95% of 5-day old females were mated, as determined by microscopy of the dissected spermatheca.

The total RNA was extracted from the mouthparts by grinding with plastic pestles, using the Direct-zol RNA Miniprep Kit (Zymo Research, Irvine, CA, USA), and treated with DNase I (Ambion, Austin, Texas, USA) with incubation for 30 min at 37 °C, according to the manufacturer’s protocols. The RNA integrity for each sample was determined using an Agilent 2100 Bioanalyzer. PolyA-tail-enriched RNA libraries were prepared by the Nevada Genomics Center using an Illumina TruSeq stranded mRNA Prep kit (Illumina Inc., Hayward, CA, USA). Paired-end sequencing was performed on an Illumina NextSeq 550 using a High Output Kit, Version 2, 150 cycles, flow cell (Illumina Inc., San Diego, CA, USA).

#### 2.2.2. Quality Control of Raw Reads

Sequence read pairs were filtered and trimmed to remove low-quality reads, adapters, and artifacts using the Trimmomatic software v0.36 with the default parameters [17]. Quality control (QC) of the reads was evaluated on each individual sample using FastQC v0.11.9 ([18], http://www.bioinformatics.babraham.ac.uk/projects/fastqc/) prior to and after trimming (22 January 2021). In addition, the FastQC reports were unified into a master report for all samples pre- and post-trimming using the MultiQC software v1.9 [19] and the RSeQC package v4.0. After the initial trimming and QC, STAR aligner v2.7.5c [20] was used to align reads to the *An. stephensi* SDA-500 genome assembly. 

The *An. stephensi* SDA-500 reference (GCA_000349045.1—VEuPathDB release #/date: release 49/05-NOV-20) and corresponding genome annotation (AsteS1.8) was downloaded from vectorbase.org. The insert sizes were calculated via PicardTools CollectInsertSizeMetrics v2.24.0 (http://broadinstitute.github.io/picard/) (22 January 2021). Following alignment, featureCounts v2.0.0 software from the subread package [21] produced the total counts, which were used to calculate the TPM (transcripts per million) values [22,23] and FPKM (fragments per kilobase of transcript per million fragments mapped) values with the R package countToFPKM v2.0.0 (DOI: 10.1093/bioinformatics/btt656). Annotation was retrieved from VectorBase on 22 January 2021.

### 2.3. Electrophysiological AsOR8 Characterization in the Drosophila Empty Neuron System

#### 2.3.1. Cloning

AsOr8 sequences were identified by using the tBLASTn function in Vectorbase [24] using the *An. gambiae* Or8 sequence as a query [8,25]. Sequences annotated as Or8 were returned from the Indian strain annotation (ASTEI08712-RA) and SDA-500 annotation (ASTE009819-RA) encoding predicted 409 or 401 amino acid products, respectively. Regardless, the 5′ and 3′ ends of the open reading frame were identical between the two sequences and the primers were designed accordingly. The primers were designed with restriction site overhangs (Forward: AsOr8F1EcoRI (ATAGAATTCACCATGCCACCAGCAAACTCTACC, Tm = 57.6 °C); Reverse: AsOr8R1XbaI (CGCTCTAGATTACTTCACATTCTTCTCATTGGGTTCG, Tm = 57.1 °C)) for cloning. 

RNA was extracted as above, from 50 heads of 5-day old *An. stephensi* females maintained on 10% sucrose, collected at 1 h prior to scotophase. cDNA was prepared using reverse transcription with the Superscript IV kit (Invitrogen, Carlsbad, CA, USA). The AsOr8 transcripts were amplified with Phusion DNA polymerase (Phusion Green HSII, ThermoFisher Scientific, Walthham, MA, USA) using a two-step PCR protocol: 98 °C for 15 s, followed by annealing at 65–56 °C (−1 °C each cycle) for 30 s and extension at 72 °C for 75 s for 10 cycles, then followed by 98 °C for 15 s, annealing at 63 °C for 30 s, and extension at 72 °C for 1 min and 15 s for 30 cycles.

The PCR products were separated by gel electrophoresis, excised, and purified using the Zymoclean Gel-DNA Recovery Kit (Zymo Research, Irvine, CA, USA). The products were digested with EcoRI and XbaI in Cutsmart buffer (New England Biolabs, Ipswich, MA, USA) at 37 °C for 2 h, followed by heat inaction of the restriction enzymes by incubating at 65 °C for 20 min. The pUAST vector was prepared for ligation following the same restriction digestion protocol [26]. For the ligation reaction, 20 fmol of digested pUAST was mixed with 80 fmol of digested insert PCR product and incubated with T4 DNA ligase (New England Biolabs) at room temperature for 1 h, followed by inactivation of the ligase by incubation at 65 °C for 10 min. The ligation product was then used to heat shock transform Top10 cells (Invitrogen, Carlsbad, CA, USA) following the manufacturer’s protocol.

The cells were plated, and the colonies were checked with PCR for the AsOr8 insert. Cells containing the AsOr8 insert were grown, and the plasmid was extracted using the Qiagen Plasmid Midi Kit (Qiagen, Germantown, MD, USA). The plasmids were Sanger sequenced (Genewiz, South Plainfield, NJ, USA) to confirm the AsOr8 sequence was cloned accurately and without introns or frameshifts.

#### 2.3.2. Drosophila Stocks and Transgenes

AsOr8 was expressed in the *Drosophila melanogaster* empty neuron system for electrophysiological characterization [27,28]. *D. melanogaster* embryos were transformed by injecting pUAST containing the AsOr8 insert (Rainbow Transgenic Flies, Inc., Camarilla, CA, USA). The transformants were crossed with a w1118;cyo/sco;Tm6/mkrs balancer line and screened for UAS-AsOr8 insertion on the third chromosome. These flies were crossed with the Δhalo line to generate a Δhalo/cyo;UAS-AsOr8/mkrs line ((wt 1118); Δhalo/cyo; 22a-Gal4/mkrs flies were kindly provided by Dr. Anandasankar Ray, University of California, Riverside, CA). The flies, which were characterized by electrophysiology, were obtained by crossing the w118;Δhalo/cyo;UAS-AsOr8/mkrs flies with w1118;Δhalo/cyo; 22a-Gal4/mkrs flies and selecting for the w118;Δhalo/Δhalo;UAS-AsOr8/ Or22a-Gal4 flies.

#### 2.3.3. Electrophysiology

Single unit extracellular recordings in the *Drosophila* empty neuron were performed on 4–14 day old Δhalo/Δhalo; UAS-AsOr8/22a-Gal4 flies and adapted from methods described before [27,29]. For all recordings, the ground electrode, a pulled glass capillary pipette filled with Ephrussi and Beadle solution [30], was slipped over an AgCl coated silver wire and placed into the fly’s right eye. For the recording electrode, a tungsten microfilament was placed into direct contact with an Ab3 sensilla. The signal was passed through an Iso-DAM8a impedance amplifier (WPI), with the high-pass filter set to 100 Hz and the low-pass filter set to 3 kHz, and digitized at 10,667 Hz using a Syntech IDAC4 Intelligent Data Acquisition Controller.

Odorants were diluted 1:100 *v*/*v* in paraffin oil and were mobilized with clean, humidified air directed toward the fly antenna at 1.4 L/min (37.5 mL/s). During stimulus presentation, a 0.5-s pulse of 3.75 mL/s amplitude was directed through the shaft of a Pasteur pipette containing 50 μL of 1:100 diluted (*v*/*v*) odorant placed on a 13-mm Whatman filter paper placed inside the Pasteur pipette. Controlled odor pulses were delivered through the odor cartridges using a Syntech stimulus controller (CS-55). Odor cartridges were used no more than three times prior to replacement with a new cartridge.

Odorants were presented to flies in sets of up to 10 odorants, including solvent control and paraffin oil (PO), while recording from a single fly sensilla. The odorant presentation order was randomized between recordings from single sensilla. Up to three sensilla per fly were used for recordings. The responses were stored and characterized using Autospike (Syntech). The action potentials were counted manually over the 500 ms period when the odor pulse passed the antenna. The average solvent response to paraffin oil was subtracted from the reported action potential firing rates.

For the mosquito capitate peg recordings, female *An. stephensi*, 3–5 days post-emergence, were fixed in a similar manner to *Drosophila*, with the ground electrode placed into the right eye of the mosquito. The maxillary palp was affixed onto a piece of double-sided tape, ventral side up, and the recording electrode was placed into contact with a capitate peg on the third or fourth palp segment. Each response to an individual odor was recorded on a different capitate peg sensilla.

### 2.4. Odorant Panel Selection

Odorants were selected to represent human and animal odors across a broad range of chemical classes, as well as heterocyclics and aromatics representative of ecologically relevant plant phytochemicals, which elicit responses from a large fraction of characterized mosquito odorant receptors (Table S1) [6,28,29]. Human and animal odors primarily consist of alcohols, aldehydes, and ketones. Carboxylic acids and amines were underrepresented in this panel, despite being potent mosquito attractants. These are not commonly perceived by dipteran Ors, although Irs respond robustly to these odorants [31].

## 3. Results

### 3.1. An. stephensi Mouthpart Transcriptome Reveals High Expression of Or8

Overall, the mapping rate (~82.5%) and mapped proper pairs (~95.1%) of the *An. stephensi* mouthpart transcripts indicated successful mapping. Furthermore, the Proper Pairs metric (~95%) confirmed these results indicating that the forward and reverse strand were properly mapped, as opposed to reverse/reverse or forward/forward mapped reads (improper pairs), which were excluded from expression quantification. STAR alignment resulted in 13,611 mapped transcripts, of which 3056 showed no expression in any of the samples. The overall alignment rate was between 81.1–83.7% (Table 1). See Table S2 for a merged file including the VectorBase annotation, TPMs, FPKMs, and feature counts.

Outside of genome-wide annotations [2], the full complement of Ors, Irs, Grs, and OBPs in *An. stephensi* has not been extensively characterized. In comparison to the more highly characterized *An. gambiae,* multiple chemoreceptor and OBP homologs were not found in *An. stephensi*, or were not annotated as such (Table 2). Several *An. stephensi* genes were annotated as unnumbered Ors, Irs, Grs, or OBPs, or as uncharacterized proteins; however, tBLASTn searches with *An. gambiae* homologs in Vectorbase revealed multiple apparent *An. stephensi* homologs (see Table S2 for full annotation list). 

Ors, Irs, and OBPs all had less annotated genes in *An. stephensi* than in *An. gambiae*, and several in each group were incompletely annotated or unannotated. Whether the reduction of genes in *An. stephensi* is a result of incomplete genome assembly or whether gene loss or duplication occurred is unclear. All Grs in *An. gambiae* were present in *An. stephensi*, although several were also incompletely annotated or unannotated.

**Figure 1:**
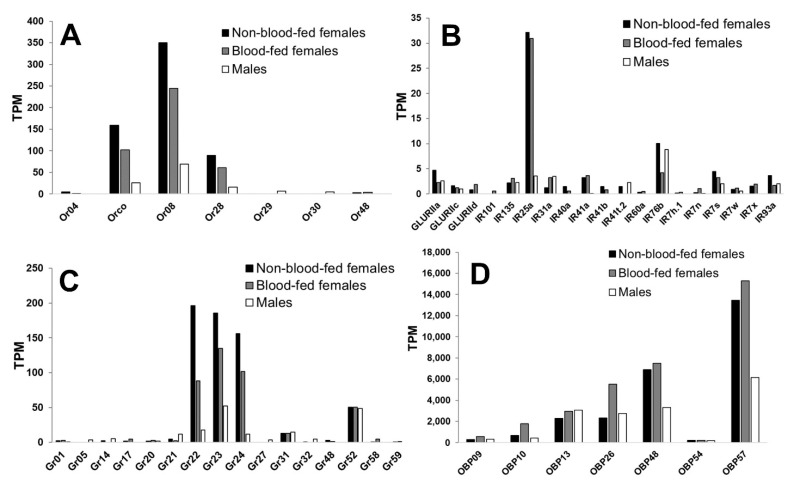
Transcript abundance of chemosensory genes. Transcript abundance of the (**A**) odorant receptors, (**B**) ionotropic receptors, (**C**) gustatory receptors, and (**D**) odorant-binding proteins in the mouthparts of *An. stephensi* non-blood-fed females (black bars), 12-h blood-fed females (gray bars), and males (white bars).

The *An. stephensi* mouthpart RNAseq (including both maxillary palpi and proboscis tissues) revealed that transcripts were detected for approximately half of the identified *An. stephensi* Ir, Gr, and OBP genes, and approximately a third of or genes (Table S2). However, a low threshold cutoff was applied at 1% of the highest TPM within each of the chemoreceptor groups or the OBPs, which resulted in approximately 7–19 transcripts detected in each group (Figure 1). The predominant Ors with high transcript abundance in *An. stephensi* mouthparts were Or7 (Orco), Or8, and Or28 (Figure 1A). Ir co-receptors Ir25a and Ir76b were also detected in the mouthparts, yet transcripts for the co-receptor Ir8a were negligible. 

A total of 17 additional Ir transcripts were detected above the 1% cutoff (Figure 1B). Gr22, Gr23, and Gr24—presumptive *An. stephensi* CO_2_ receptor orthologs [25]—were highly expressed in the mouthparts. Gr31 and Gr52 were also consistently expressed, at lower abundance, in addition to trace detection of other Grs (Figure 1C). The most abundant odorant-binding proteins expressed were OBP13, OBP26, OBP48, and OBP57 and, to a lesser degree, OBP9, OBP10, and OBP54 (Figure 1D). The transcript abundance between sexes or by feeding status was proportionally similar, with less expression overall detected in males. However, the collection of single replicates for sex/feeding status prevented statistical comparison of the expression levels.

### 3.2. AsOr8 Is Receptive to Alcohols and Ketones in the Human Volatile Spectrum

**Figure 2:**
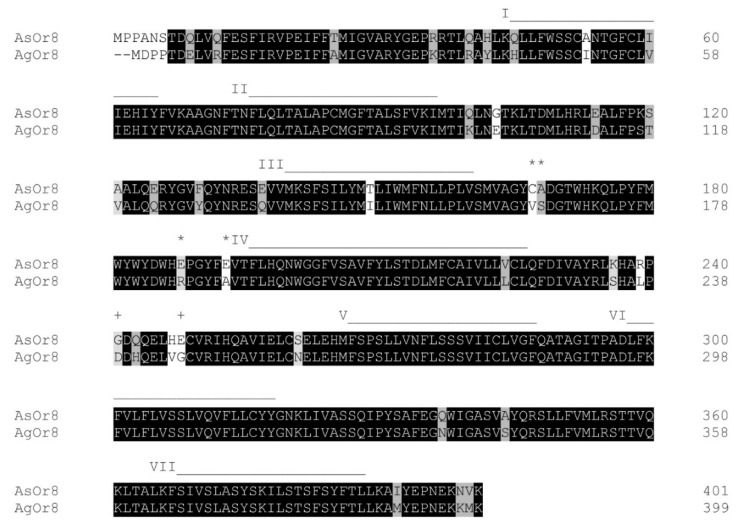
Primary structure comparison of AsOR8 and AgOR8. A: Amino acid alignment of AsOR8 and AgOR8. Highlighted areas indicate identical and conserved residues as designated by ClustalOmega [32]. Black = identical residues; dark gray = strongly similar residues; and light gray = weakly similar residues (for amino acid similarity groups, see: http://www.clustal.org/download/clustalx_help.html) (accessed on the 8 February 2021). Predicted transmembrane (TM) domains I–VII are indicated as a line above the alignment (N-terminus is cytosolic). “+” above the sequence indicates an amino acid change in the sequence determined in this study from the predicted *An. stephensi* SDA-500 genome sequence. Differences in extracellular loop 2 between species are noted with “*”.

To further understand the role of the highly expressed AsOr8 in the mouthparts, we explored the sensitivity of this receptor to assorted odorants by expressing it in the *Drosophila* empty neuron system. This is an in vivo expression system in the fly antenna, in which individual Ors (from *Drosophila* or a mosquito species) can be expressed in a mutant neuron that lacks an endogenous functional Or [6,27,29]. The cloned AsOr8 sequence encoded a 401 amino acid protein nearly identical to the *An. stephensi* SDA-500 annotation, but with substitutions at positions 241 (Asp to Gly) and 248 (Ala to Glu) (GenBank# MW076538). The cloned sequence is 89.5% identical to *An. gambiae* Or8 (AgOr8) ([25]; Accession: AGAP001912) (Figure 2). Extracellular loop regions were identical between AsOr8 and AgOr8 except for four amino acid differences in extracellular loop 2 (Figure 2).

**Figure 3:**
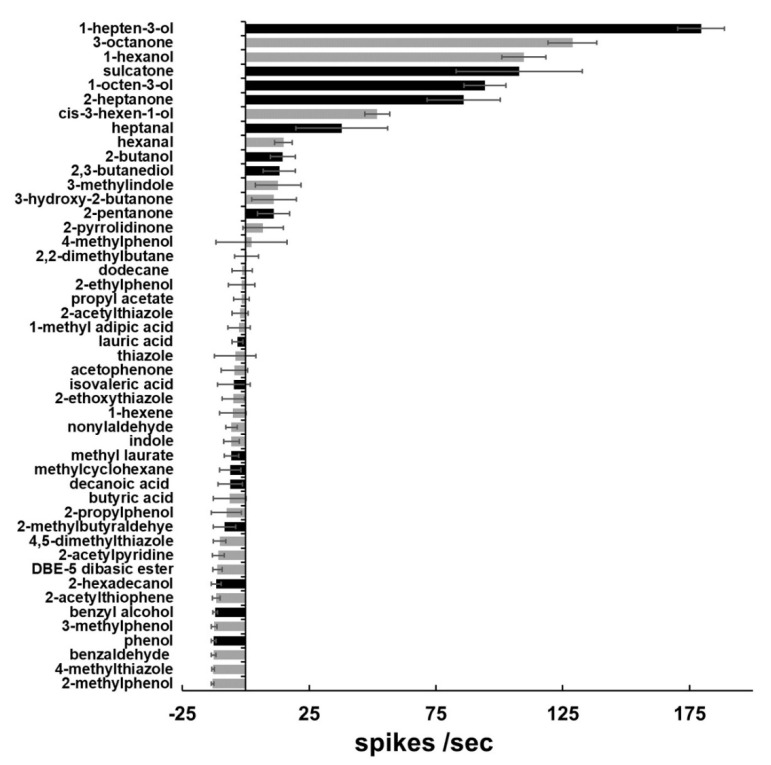
Responses of the *Drosophila* empty neuron expressing AsOr8 to selected volatiles. Response of AsOr8 to selected volatiles, diluted 1:100 (*v*/*v*) in paraffin oil, in the *Drosophila* empty neuron system. Black bars: odorant detected in human emanations. Gray bars: odorant not detected in human emanations (see Table S1 for responses to all compounds tested).

**Figure 4:**
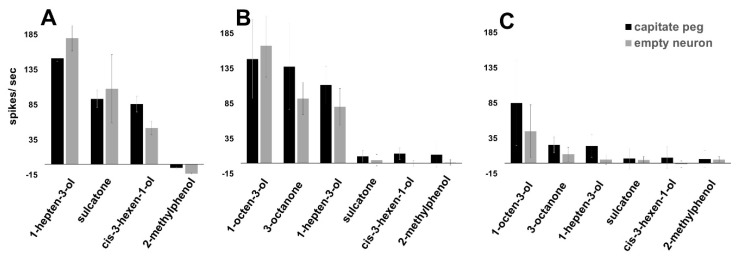
The *An. stephensi* cpB neuron responds to the same odorants as the *Drosophila* empty neuron expressing AsOr8. Responses of the *An. stephensi* maxillary palp cpB neuron (black bars) or the *Drosophila* empty neuron expressing AsOr8 (gray bars) to odorants that elicited the strongest responses, at 10^−2^ (**A**), 10^−4^ (**B**), and 10^−6^ (**C**) (*v*/*v*) dilutions in paraffin oil. Recordings were taken from unique capitate peg sensilla on female *An. stephensi* maxillary palps or AB3 sensilla on *Drosophila* antenna. CpB counts were taken within the 500 ms window as the odor pulse passed over the *An. stephensi* maxillary palps or the antennae. At 10^−2^, the cpB neuron was activated strongly by 1-octen-3-ol and 3-octanone; however, the initial spike amplitude became indistinguishable from background noise within the counting interval.

As expressed in the *Drosophila* empty neuron system, AsOr8 was sensitive to 6–8 carbon alcohols and ketones, with 1-hepten-3-ol eliciting the greatest activity at high concentrations. 3-octenol, 1-hexanol, sulcatone (6-methyl-5-hepten-2-one), 1-octen-3-ol, and 2-heptanone also elicited strong responses (Figure 3 and Figure 4A). At lower odorant concentrations (10^−4^, 10^−6^ *v*/*v* dilutions), AsOr8 exhibited the greatest sensitivity to 1-octen-3-ol, and was also sensitive to 3-octanone and 1-hepten-3-ol; however, the response to sulcatone was insignificant (Figure 4B,C).

### 3.3. Maxillary Palp Capitate Peg Recordings Mirror AsOr8 Recordings

To explore the odor responsiveness of the mouthparts in vivo, single sensilla electrophysiological recordings from female *An. stephensi* capitate pegs located on the maxillary palps were performed. Although the specific expression distribution of AsOr8 in capitate pegs was unavailable, the capitate peg ‘B’ (cpB) neuron responses mirrored those taken from the empty neuron in *Drosophila* exogenously expressing AsOr8 (Figure 4B,C). The cpB neuron responded strongly to 1-hepten-3-ol, sulcatone, 1-octen-3-ol, and 3-octanone at high concentrations (10^−2^) (Figure 4A), while exhibiting the greatest sensitivity towards 1-octen-3-ol. For each odorant tested at lower concentrations, the cpB neuron exhibited a greater response than the *Drosophila* empty neuron expressing AsOr8, with the exception of 1-octen-3-ol (Figure 4B,C).

## 4. Discussion

The proboscis and maxillary palps play a crucial role in the host-seeking behavior of female mosquitoes, as was demonstrated in *An. stephensi* [14]. Here, we report on the first *An. stephensi* mouthpart transcriptome to more fully explore the chemosensation of this important set of sensory appendages. Notably, transcripts for AsOr8 were highly abundant, and we further characterized this receptor to better understand its potential chemosensory role, particularly with regard to host-seeking and volatiles found in the human odor spectrum.

In mosquitoes, Or8 has a conserved role in detecting (R)-1-octen-3-ol [6,25,33,34,35]. In blood-feeding species, Or8 is also activated by several compounds emitted by vertebrates, such as 1-hepten-3-ol, sulcatone, and 2-heptanone [6,7,25]. Curiously, the ability to detect (R)-1-octen-3-ol is retained in *Toxorhynchites ambionensis* Or8; however, in this species, which does not does not seek blood meals, the receptor was much more narrowly tuned. It was not responsive to 1-hepten-3-ol, 2-heptanone, or 3-octanone, and it is suspected to function in detecting plant volatiles [35].

In the current study, AsOr8 responded to similar vertebrate-emitted odorants to the homolog of its closest characterized relative, AgOr8 with the exception of sulcatone, which activated AsOr8 in our study, but did not elicit a strong response from AgOr8 [6,7,25]. Functionally, the activation of AsOr8 by sulcatone (Figure 3) and the converse apparent unresponsiveness of AgOr8 to this compound are interesting in light of this compound’s role in human host selection in *Ae. aegypti* [36]. These receptors share high amino acid conservation; however, specific amino acid changes in Or8 may be responsible for the difference in response of AsOr8 and AgOr8 to sulcatone (Figure 2). 

Although the key amino acids for the odorant selectivity of Or8 have not been characterized, a mutation screen of AgOrs 13 and 15 suggested that changes to amino acids in extracellular loops 2 and 3 and transmembrane regions V–VII shifted the odorant specificity in these receptors [37]. These regions in AsOr8 and AgOr8 were identical except for four amino acid changes in extracellular loop 2 (Figure 2) and are possible candidates for a structural basis for the odor sensitivity shift between these species’ receptors. 

However, the b neuron of the *An. gambiae* capitate peg is responsive to sulcatone, to a slightly lesser degree compared with the b neuron of *An. stephensi* [25]. This raises the possibility that structural differences between these receptors may be compensated for by other components of the OSN environment, such as OBPs. In addition, several AgOrs do display some sensitivity to sulcatone, including AgOr30 [38], AgOr39 [7,38], AgOr57, AgOr75 [6], and the maxillary palp-expressed AgOr28 [7,25]. Further characterization of *An. stephensi* may demonstrate redundancy of sulcatone detection in other Ors also.

The cpB neuron and the fly empty neuron expressing AsOr8 were both most responsive to 1-octen-3-ol, 1-hepten-3-ol, and 3-octanone at the lowest odorant concentrations tested (Figure 4B,C). In the natural environment, insects encounter individual odorants at low concentrations within complex mixtures. The detection of these odorants and structurally similar molecules is, therefore, likely to be the most biologically relevant function of the cpB neuron and AsOr8. Importantly, both 1-hepten-3-ol and 1-octen-3-ol are higher abundance components of human odors [39]. Comparison of the AsOr8 responses expressed in the *Drosophila* empty neuron system to the cpB neuron of *An. stephensi* maxillary palpi showed similar responses to odorants yet with an increased firing rate in the cpB neuron readings. 

The increase in the apparent sensitivity of the cpB neuron to some odorants could be attributed to differences in the interaction of AsOr8 with Orco of *Drosophila* and *An. stephensi* but could also be influenced by other differences between the neuronal environments, including differences between secreted soluble proteins, such as OBPs. On the maxillary palps of *Anopheles* mosquitoes, there is only one sensilla type—the capitate peg. On the *An. gambiae* maxillary palps, the capitate peg sensilla houses the CO_2_ detecting neuron, which expresses Gr22, Gr23, and Gr24, paired with two odor sensory neurons expressing Or8 and Or28 [25]. The RNA-seq and electrophysiology results presented in this study support a conserved neuronal arrangement in the *An. stephensi* capitate pegs.

In addition to our characterization of AsOr8, comparing the *An. stephensi* mouthpart transcript abundance of chemosensory receptors and OBPs to previously published *An. coluzzii* and *An. quadriannulatus* maxillary palpi transcriptomes [8,11] showed broad similarity with some exceptions. The high expression of the Orco, Or8, and Or28; Ir25a and Ir76b; and Gr22-24 homologs in *An. stephensi* is consistent with the maxillary palpi expression of other anophelines [8,11,25,40]. Ir7s, Ir31a, Ir93a, and Ir135, notably, had a greater transcript abundance by proportion in *An. stephensi* compared with in other anophelines. Conversely, Ir75k is more highly expressed in *An. coluzzii* and *An. quadriannulatus*. 

Ir100a is prominently expressed in both *An. coluzzii* and *An. Quadriannulatus;* however, *An. stephensi* apparently lacks this homolog. Gr52 is expressed in the mouthparts of all species except for *An. quadriannulatus* and has proportionately high expression in both males and females. However, Gr31 was expressed in *An. stephensi* but was not detected in the maxillary palpi of other anophelines. OBP expression follows a conserved pattern in anophelines with high expression of OBP 48 and 57 and minor expression of OBP10 and 25. A direct homolog for AgOBP25 was not found in *An. stephensi*, but instead the detectable expression of AsOBP26 may suggest a functional, if divergent, homolog. The OBP13 expression was notably higher in *An. stephensi* when compared to other anophelines.

It is interesting to speculate on the differing expression patterns between *An. stephensi* and Anophelines from prior studies as it relates to species-specific odor perception and attraction. However, some caution is warranted. These differences may be attributable to differences in study conditions, such as age and time of day, and the precise tissues examined (maxillary palps versus all structures within the mouthparts). For instance, expression in the labellum of *An. coluzzii* varied markedly from expression in the maxillary palpi of this species [41]. 

This may explain the apparent differential expression of OBPs 13 and 54 in *An. Stephensi,* which were not detectably expressed in *An. coluzzii* or *An. quadriannulatus* maxillary palpi transcriptomes, while OBPs 14 and 54 were expressed in the labium of *An. coluzzii* [41]. In addition, *An. stephensi* apparently lacks the homologs described in the archetypical *An. gambiae*, although whether this is a result of gene loss in *An. stephensi*, gene duplication in the *An. gambiae* lineage, or incomplete *An. stephensi* genome assembly remains to be determined. A recent genome reassembly of *An. stephensi* reported 54 Ors [42], in contrast to the 59 apparent Ors that we identified in the current study. A thorough re-annotation of chemoreceptors, and the OBPs particularly, may be warranted. Further investigation will be required to elucidate whether the transcriptome generated in the current study accurately reflects consistent expression differences between species or under different physiological states.

## 5. Conclusions

Overall, our study provides an initial exploration into the expressed chemosensory repertoire of the mouthparts of adult *An. stephensi*, an important malaria vector in South Asia. AsOr8 is a highly expressed component in the mouthparts and is sensitive to 1-octen-3-ol as well as several human-emitted odorants. Interestingly, characterization of this receptor suggested differences in the detection of sulcatone, a component of the human volatile spectrum, compared to a homolog in the well-studied model malaria vector, *An. gambiae,* and could lay the foundation for further characterizing the structure–function relationships of mosquito odor receptors. Further exploration of the chemosensory protein expression in *An. stephensi*, particularly in the antennae, may highlight differences that elucidate the species- or strain-specific dynamics of host-seeking and, importantly, human host choice, a behavior that strongly impacts human disease transmission.

## Figures and Tables

**Table 1:** RNAseq mapping quality to the *An. stephensi* SDA-500 genome assembly.

Sample	Total Read Pairs	Mapped Reads	Overall Alignment Rate	Proper Pairs	Fragment Length	Total Feature Counts	Total FPKMs
Blood-fed female	54,257,482	45,434,941	83.7%	94.9%	171	29,106,087	1,493,597
Non-fed female	47,808,166	38,786,401	81.1%	95.1%	179	25,379,805	1,382,585
Male	36,364,670	30,038,819	82.6%	95.2%	152	20,034,497	1,279,250

**Table 2:** *An. stephensi* Or, Ir, Gr, and OBP genes corresponding to annotated *An. gambiae* homologs.

Chemosensory Genes	Or	Ir	Gr	OBP
Total *An. stephensi* chemosensory genes (number less than *An. gambiae*)	59 (17)	37 (12)	60 (0)	42 (24)
Unnumbered or unannotated	17	14	8	35
Expression detected	18	22	35	30

## Supplementary Materials

The following are available online at https://www.mdpi.com/article/10.3390/insects12070593/s1, Table S1: AsOr8 response to odorant panel: Spikes/sec (±SD) of all selected odorants tested against AsOr8 as expressed in the *Drosophila* empty neuron system. Odorants that have a human or animal association are noted. Table S2: RNAseq data for chemoreceptors and OBPs: TPMs and feature counts of *An. stephensi* Ors, Irs, Grs, and OBPs. Vectorbase Gene IDs are listed for all *An. stephensi* genes. Red text in the Gene Name column indicates that no *An. stephensi* ortholog to an *An. gambiae* gene was found.
